# Filamentous bulking caused by *Thiothrix* species is efficiently controlled in full-scale wastewater treatment plants by implementing a sludge densification strategy

**DOI:** 10.1038/s41598-017-01481-1

**Published:** 2017-05-03

**Authors:** Olivier Henriet, Christophe Meunier, Paul Henry, Jacques Mahillon

**Affiliations:** 1 Laboratory of Food and Environmental Microbiology, Université catholique de Louvain, Louvain-la-Neuve, Belgium; 2 CEBEDEAU, Research and Expertise Center for Water, Allée de la découverte, 11 (B53), Quartier Polytech 1, B-4000 Liège, Belgium

## Abstract

Filamentous bulking caused by *Thiothrix* species is responsible for sludge washout and loss of performance in dairy wastewater treatment plants. A long-term study was conducted over 1.5 years to test three different mitigation strategies in a full-scale plant composed of two parallel sequential batch reactors (SBR1 and 2). Strategies based on polyaluminium chloride addition and volatile fatty acids reduction were ineffective to permanently solve the problem. On the contrary, modification of the reactor cycle based on the implementation of a periodic starvation proved efficient to solve the biomass wash-out and drastically reduce the sludge volume index in both reactors. Bacterial diversity analysis using 16S amplicon sequencing and quantitative PCR indicated a reduction of *Thiothrix* abundance from 51.9 to 1.0% in SBR1 and from 71.8 to 0.6% in SBR2. Simultaneously, the abundance of the glycogen-accumulating bacterium *Candidatus* Competibacter increased in both reactors. Microscopy analysis confirmed the transition between a bulking sludge towards a granular-like sludge. This study confirms the applicability of a periodic starvation to (1) solve recurring *Thiothrix* bulking, (2) convert loose aggregates into dense and compact granular-like structures and (3) considerably reduce energy demand for aeration.

## Introduction

Filamentous bulking is a major cause of activated sludge process deficiency. It arises from an overgrowth of filamentous organisms and induces important losses of biomass that considerably reduce the removal efficiency of suspended solids, chemical oxygen demand (COD) and nutrients. The challenge in preventing filamentous bulking resides in the large diversity of filamentous organisms and their specific ecology. In particular, filamentous bacteria phylogenetically related to the gamma-proteobacterium *Thiothrix* thrive in wastewaters characterized by high organic loading rates, high concentrations of low molecular weight fatty acids and reduced sulfur compounds, low dissolved oxygen (DO) concentrations and nutrient deficits^1–3^.

Dairy effluents are characterized by high concentrations of lactose, fats and proteins that contribute to high COD contents. Wastewaters from cheese manufacturing factories have the highest COD concentrations and often present a deficit in nitrogen^4^. Butter production releases wastewaters with particularly high concentrations of fats, contributing to a high COD content^5^ while ice-cream wastewaters tend to have a lower, more biodegradable, COD content^6^. Since oxygen consumption in the treatment plant is often kept to a minimum by most industrials to reduce energy costs, COD-rich dairy wastewaters frequently lead to the growth of filamentous bacteria^7^. Additionally, unbalanced ratio of nitrogen, phosphorus and readily biodegradable COD (rbCOD) is susceptible to favor the overgrowth of *Thiothrix* species.

Strategies to control filamentous bulking can be either unspecific or specific. Unspecific methods involve the use of chemicals (chlorine, ozone, H_2_O_2_, iron or aluminium salts) to kill indistinctively microorganisms protruding from the flocs, including filamentous microbes. They often prove unsatisfactory when the bulking episodes are recurring due to their transient effect on the activated sludge ecosystem. Specific methods include procedures that modify the conditions encountered in the reactor by the biomass to create a hostile environment for the targeted filamentous organisms. Although these strategies require a preliminary identification of the microbial species involved in the bulking event, they are usually preferable as they can permanently solve the process dysfunction. A common setup in wastewater treatment plants consists in installing an anaerobic plug-flow selector ahead of the biological treatment to provide the biomass with a high food-to-microorganisms ratio. This system is meant to favor microorganisms having high rate kinetics for substrate uptake and a metabolic ability to accumulate internal storage products^8^. Ideally, the anaerobic selector must be followed by a fully aerated reactor performing a complete substrate oxidation to give an ecological advantage to microorganisms holding storage products. The presence of an anaerobic tank ahead of a completely mixed aerobic reactor was previously reported to limit/suppress *Thiothrix*-caused bulking in dairy wastewater treatment plants^9^. While *Thiothrix* remained physiologically active under prolonged anaerobic conditions, it seemed that its growth was limited in such environments^10^.

Process configurations involving successive anaerobic and aerobic reactors are restricted to continuous flow systems but the operational scheme can be implemented in sequential batch systems by alternating anaerobic and aerobic phases. In sequential batch reactors (SBRs), this strategy is commonly named the “feast and famine” regime or “periodic starvation” and is specifically used in aerobic granulation^11^. A periodic starvation strategy was recently applied in a lab-scale SBR fed with a dairy wastewater to convert a bulking sludge caused by *Thiothrix* and *Leptothrix* species into a well-settling granular sludge^12^. This procedure resulted in the decline of the filamentous bacteria and the growth of the phosphate accumulating organism (PAO) *Candidatus* Accumulibacter and the glycogen accumulating organism (GAO) *Candidatus* Competibacter. These accumulating bacteria are particularly interesting since they contribute to increase the overall density of the sludge (a phenomenon called “sludge densification”) by building dense internal inclusions of glycogen, polyhydroxyalcanoate (PHA) or polyphosphate^13^.

Based on these previous lab-scale experiments, the present study aimed to implement a sludge densification strategy in an industrial wastewater treatment plant treating dairy wastewaters and experiencing a recurring *Thiothrix*-based filamentous bulking. The plant was composed of two parallel SBRs fed by an influent pre-treated in a dissolved air flotation (DAF) unit. The sludge densification was achieved by applying a feast-and-famine regime in both SBRs. *Thiothrix* abundance was followed by molecular tools and the overall performances of the plant were followed over a period of 1.5 years.

## Results and Discussion

**Figure 1:**
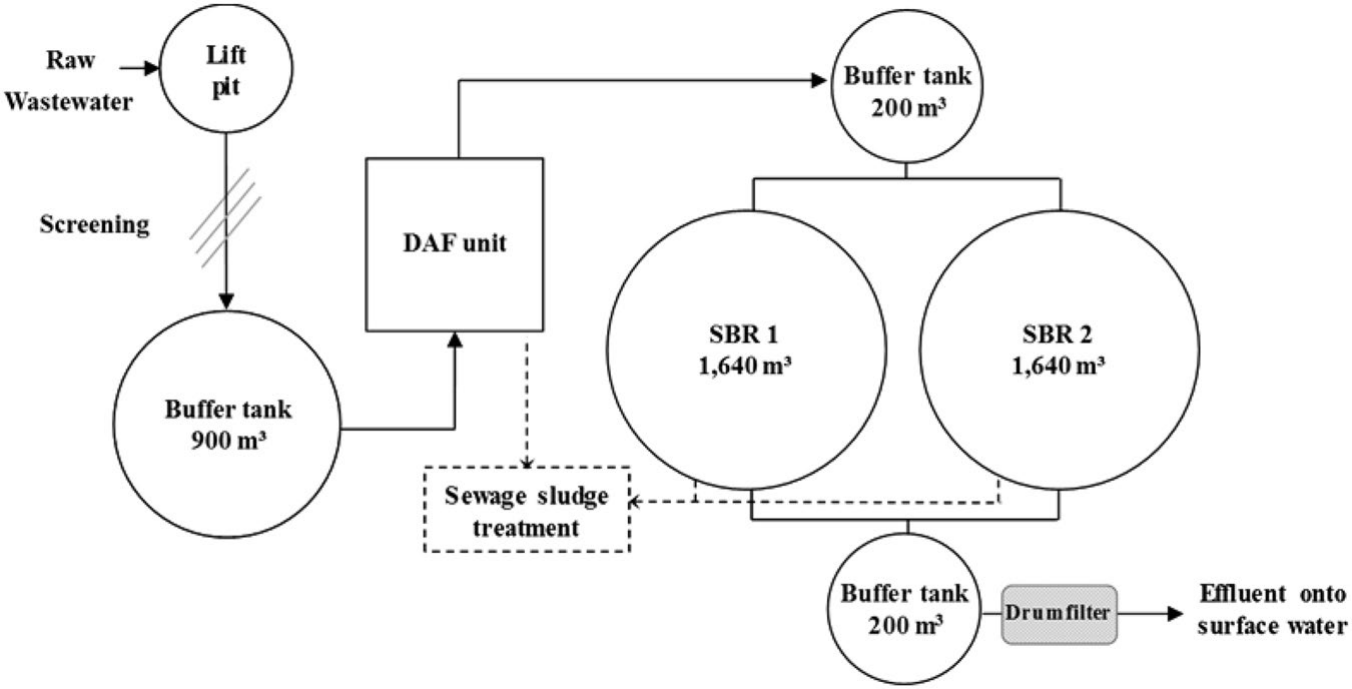
Flow diagram of the dairy wastewater treatment plant. The raw wastewater was screened then collected in an equalization buffer tank. During production, a second buffer tank received a continuous flow from the dissolved air flotation (DAF) unit. The wastewater was then treated with two conventional sequential batch reactors (SBR1 and SBR2) operated in parallel. The effluent was collected in a buffer tank, screened through a drum filter and released in the receiving watercourse.

Recurring filamentous bulking caused by *Thiothrix* species was experienced by a wastewater treatment plant (WWTP) treating the process water from the dairy factory Luxlait (Roost/Bissen, Luxembourg). These events were responsible for an increase of the total suspended solids (TSS) in the effluent. The operator adapted the plant with a drum filter downstream from the biological stage (Fig. 1) to reduce the TSS in the effluent and started a non-specific chemical procedure to reduce the population of *Thiothrix*.

### Polyaluminium chloride addition (Strategy I)

**Figure 2:**
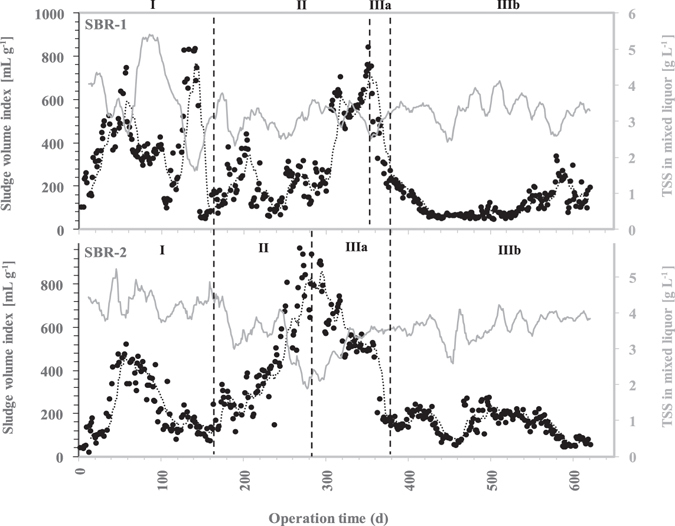
Sludge volume index (SVI, •) profile and TSS in mixed liquor (grey lines) of SBR1 (top) and SBR2 (bottom) over the experimental period. Mitigation strategies consisted of the addition of polyaluminium chloride (I), inhibition of VFA production (II) and modification of the feeding pattern with (IIIa) or without (IIIb) aeration in the buffer tanks. SVI values were measured after 30 min settling. Day 1 corresponds to January 1^st^, 2015.

The non-specific strategy was applied from early February 2015 (day 41) on both SBRs and lasted until day 63. During the whole period, the SBRs were fed aerobically during 2 h (Table 1). The addition of polyaluminium chloride proved inefficient and the overgrowth of *Thiothrix* could not be solved. The SVI remained high during the whole period of treatment. In SBR1, two SVI peaks were recorded: the first peak reached 428 mL g^−1^ around day 98 and the second peak went above 800 mL g^−1^ around day 140 (Fig. 2). A washout of the biomass was observed and the TSS in the mixed liquor went down to 1.5 g L^−1^ after the second peak. Consequently, the quality of the effluent was mainly degraded by the TSS concentration and the overall removal efficiency of both SBRs was limited to 89 ± 9%, 71 ± 12% and 67 ± 12% for total COD, total nitrogen (TN) and total phosphorus (TP), respectively (Supplementary Figure S1). At the end of this period, the biomass maintained an important proportion of *Thiothrix* that created a net-like structure, bridging the flocs together and reducing the bulk density of the sludge (Supplementary Figure S2). The sludge from SBR2 was not as deeply impacted by the filamentous bulking as observed in SBR1. The SVI also reached high values (around 525 mL g^−1^) around day 60 but the initial settleability of the sludge was regained at around day 100. A microscopic observation of the sludge revealed a lesser amount of interconnecting filaments and the presence of small floccular aggregates (Supplementary Figure S2).

**Table 1:** Operational parameters applied to the full-scale SBRs (SBR1 and SBR2). Mitigation strategies consisted of the addition of polyaluminium chloride (I), inhibition of VFA production (II) and modification of the feeding pattern with (IIIa) or without (IIIb) aeration in the buffer tanks.

**SBR-1**					
**Strategy**		**I**	**II**	**IIIa**	**IIIb**
Days		1–165	166–357	358–384	385–621
**Buffer tanks**					
Mixing with aeration		No	Yes	Yes	No
**Cycle**					
Aerobic feed	min	120		0	
Anaerobic feed	min	0		95	
Aeration	min	190		210	
Settling	min	50–60		40	
Decanting	min	110		115	
Sludge purge and idle	min	10		20	
**SBR-2**					
**Strategy**		**I**	**II**	**IIIa**	**IIIb**
Days		1–165	166–282	283–384	385–621
**Buffer tanks**					
Mixing with aeration		No	Yes	Yes	No
**Cycle**					
Aerobic feed	min	120		0	
Anaerobic feed	min	0		95	
Aeration	min	190		210	
Settling	min	50–60		40	
Decanting	min	110		115	
Sludge purge and idle	min	10		20

### Inhibition of volatile fatty acids production in the buffer tanks (Strategy II)

Considering the persistence of *Thiothrix* in SBR1, a complete diagnosis of the process was conducted to identify the potential causes of the bulking. Two factors were assumed to play a major role in the growth of *Thiothrix*: the presence of volatile fatty acids (VFA) combined with a high aerobic fill time ratio (FTR) and a relatively low DO (1.4 to 4.0 mg L^−1^). The possible ways of improvement were limited: the influent composition could not be easily adapted, a significantly lower aerobic FTR would involve costly capital expenditure (electromechanical and piping modifications) and the DO in the SBRs had to remain low to avoid excessive operational expenditure. VFA were hypothesized to arise from a hydraulic retention time (HRT) of around 15 h in the buffer tanks upstream from the biological treatment that caused the development of acidogenic bacteria. Consequently, the strategy adopted was the installation of an aerator in the buffer tanks upstream from the SBRs to slightly increase the DO. This modification was applied from day 166 until day 384. Although the average VFA load decreased from 0.144 to 0.092 kg COD_VFA_ m^−3^ d^−1^, it remained sufficient to favor the overgrowth of *Thiothrix*, which uses readily biodegradable substrates^14^. According to the “kinetic selection” theory^15^, filamentous microorganisms are characterized by a maximum growth rate (μ_max_) and affinity constant (K_s_) lower than the floc-forming microorganisms. Consequently, the low COD_VFA_ concentration (10 ± 8 mg O_2_ L^−1^) during the aerobic feeding phase led to a higher specific growth rate for *Thiothrix* compared to floc-forming bacteria. The sludge SVI from SBR2 increased steadily over the first 116 days of operation until it reached 950 ml g^−1^ (Fig. 2). The washout was responsible for a dramatic decrease of the biomass concentration from 4.1 to 1.7 g L^−1^ within 37 days (day 245 to 282). At day 282, the sludge was dominated by filamentous organisms that reduced the bulk density (Supplementary Figure S2). Even though the sludge quality remained poor, removal efficiencies of 96 ± 4% of the total COD, 83 ± 7% of TN and 88 ± 5% of TP were achieved during this period due to the presence of a drum filter polishing unit (Supplementary Figure S1).

### Densification by periodic starvation (Strategy III)

It was therefore decided to change the approach by modifying SBR2 cycle while maintaining the aeration in the buffer tanks. The feed was provided under unaerated conditions (feast) during 95 min followed by an extended aeration period of 210 min (famine). This procedure was intended to avoid VFA consumption by *Thiothrix* and simultaneously favor the growth of accumulating microorganisms able to store biopolymers, mainly polyhydroxyalcanoate (PHA) under anaerobic conditions^16^.

This new protocol was not applied on SBR1 until day 357 because the sludge characteristics were not degraded enough to risk a joint modification of both SBRs. However, on day 357 the SVI had reached almost 800 mL g^−1^ and the same cycle modification was also applied to SBR1. After two weeks, this feast and famine regime was able to maintain the residual concentration of COD_VFA_ and soluble COD (mainly soluble substrates such as lactose) at the end of the anaerobic phase below 1 mg O_2_ L^−1^ and 40 mg O_2_ L^−1^, respectively. This feeding pattern improved the SVI of SBR2 from 950 to 200 mL g^−1^ in 90 days and the biomass concentration stabilized around 3.5 g L^−1^ (Fig. 2). The effect of this cycle modification on SBR1 was even more pronounced: the SVI went from 800 to 80 mL g^−1^ in 60 days (day 358 to 418) while the TSS slightly increased to reach 3.4 g L^−1^. The settleability of the sludge from both reactors was maintained and SVI peaks were no longer observed until the end of the experiment. The aeration in the buffer tanks was stopped at day 385 to verify the stability of the sludge despite a potential installation of acidogenic metabolisms upstream from the biological treatment (Strategy IIIb). The SVI and biomass concentration remained unchanged in both reactors, which proved that the feast-famine regime was sufficient by itself to solve the bulking event. To highlight additional benefits of the densification strategy, the drum filter unit was by-passed from day 418 to day 461. Interestingly, the removal efficiencies for total COD, TN and TP remained stable at 96 ± 3%, 88 ± 7% and 93 ± 7%, respectively (Supplementary Figure S1).

**Figure 3:**
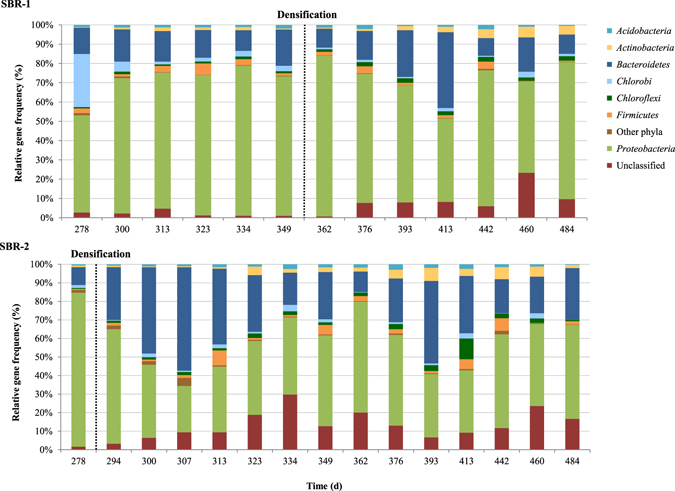
Distribution of the bacterial phyla in SBR1 (top) and SBR2 (bottom) between days 278 and 484. The abundance of each phylum is expressed as the relative gene frequency of the corresponding OTUs.

**Figure 4:**
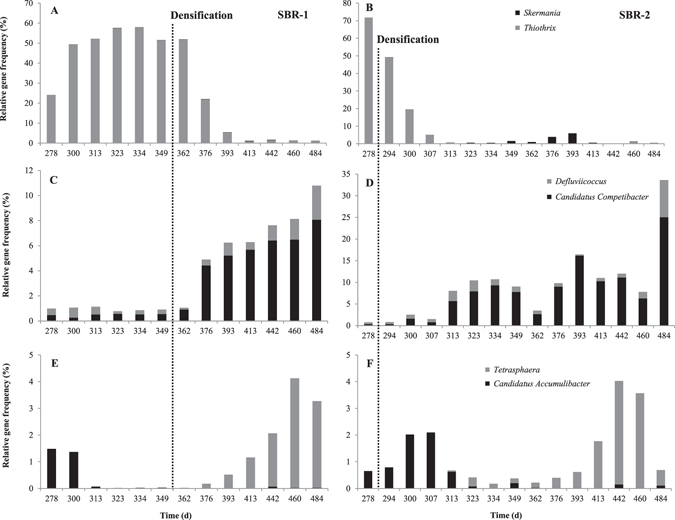
Temporal change in the relative gene frequency in SBR1 (left) and SBR2 (right) of *Skermania* spp. and *Thiothrix* spp. (**A** and **B**), *Defluviicoccus* spp. and *Candidatus* Competibacter (**C** and **D**), *Tetrasphaera* spp. and *Candidatus* Accumulibacter (**E** and **F**).

The effect of the cycle modification was analyzed by microscopy and molecular tools from day 250 to 500 to assess changes in the bacterial diversity and the impact on *Thiothrix* and PHA-accumulating bacteria (PAO and GAO). Although fed with wastewater coming from the same buffer tank, the distribution of the bacterial phyla in the reactors was different (Fig. 3). The initial bacterial diversity (day 278) of the sludge from SBR1 contained principally OTUs associated to the phyla *Proteobacteria*, *Chlorobi* and *Bacteroidetes* while the sludge from SBR2 was principally composed of OTUs associated to the phylum *Proteobacteria* (83.1%). The overrepresentation of this phylum was mostly due to the abundance of *Thiothrix*-related OTUs (71.8%). More generally, the variation of *Proteobacteria* abundance over time in both reactors was mainly driven by modifications of *Thiothrix* abundance (Fig. 4A,B). The residual diversity was shared between phyla *Bacteroidetes* and to a lesser extent *Actinobacteria*. The OTUs affiliated to *Actinobacteria* were largely associated to the PAO *Tetrasphaera* ^17^ and the OTUs associated with *Bacteroidetes* were mostly affiliated to the order *Sphingobacteriales* that includes members regularly identified in activated sludge^18^.

**Figure 5:**
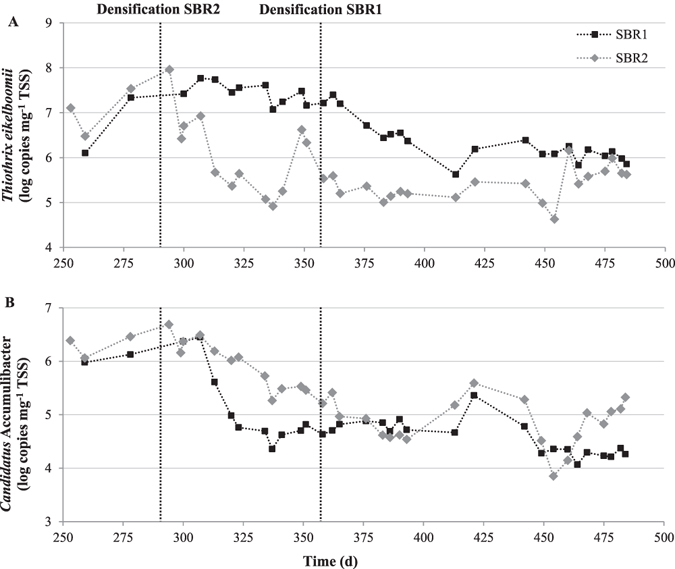
Temporal change in the concentration of *Thiothrix eikelboomii* (**A**) and *Candidatus* Accumulibacter (**B**) expressed in number of copies of 16S rRNA operon per mg TSS, in SBR1 and SBR2 from day 250 to 500. Densification strategy (Strategy III) was applied from day 283 on SBR2 and from day 358 on SBR1.

Bacterial diversity analysis revealed the combined presence of two species from the genus *Thiothrix*: *Thiothrix eikelboomii* and *Thiothrix flexilis*. Both species evolved with a similar pattern, although not with identical gene frequencies. At the beginning of Strategy III (day 278), the relative gene frequency of both species reached a total of 71.8% in SBR2 (Fig. 4B). The qPCR quantification of *T. eikelboomii* indicated that its concentration was high, with a number of 16S rRNA gene copies of 7.5 log mg^−1^ TSS (Fig. 5A). The effect of the densification on *Thiothrix* abundance was fast and its gene frequency went down to 0.6% only 30 days after the start of Strategy III. The concentration of *T. eikelboomii* was minimal on day 337 with a number of copies of 4.9 log mg^−1^ TSS. A short-term recrudescence (6.6 log copies mg^−1^ TSS) was observed around day 349 but its concentration quickly regained a low and stable value until the end of the experiment. The densification strategy had a similar effect on SBR1. The relative gene frequency of genus *Thiothrix* steadily declined shortly after the beginning of Strategy III from 51.9 to 1.0% between days 362 and 413 (Fig. 4A). The abundance of *T. eikelboomii* decreased from 7.4 to 5.6 log copies mg^−1^ TSS during the same period and stabilized around 6.0 log copies mg^−1^ TSS until the end of the experiment (Fig. 5A). The microscopic observation of the sludge confirmed the absence of filaments and indicated the presence of dense clusters with granular-like architecture in both reactors (Supplementary Figure S2). The elimination of *Thiothrix* in SBR2 was accompanied from days 323 to 413 by the growth of another filamentous bacterium associated to the genus *Skermania* (Fig. 4B). This pine tree-like bacterium caused the apparition of foam at the reactor surface but did not impact the sludge settleability. The absence of *Skermania* in SBR1 speaks in favor of a localized phenomenon unrelated to external factors and it could not be related to any specific event.

Beyond the reduction of *Thiothrix* abundance in the reactors, the cycle modification also aimed to favor the development of bacteria able to store intracellularly PHA. Specifically, two groups of bacteria were targeted: GAO and PAO. Both were reported to help structuring the biomass into dense and stable aggregates (*i.e*. granules)^19, 20^. Most common GAO reported in the literature were the gamma-proteobacterium *Candidatus* Competibacter and the alpha-proteobacterium *Defluviicoccus* ^21, 22^. Although *Defluviicoccus* was initially present with a similar gene frequency as *Ca*. Competibacter on day 278 in SBR1 (0.5% for both genera) and SBR2 (0.3 and 0.5% for *Ca*. Competibacter and *Defluviicoccus* respectively), the gene frequency of *Ca*. Competibacter became dominant soon after the beginning of the densification strategy (Fig. 4C). This change was explained by a strong response of *Ca*. Competibacter to the cycle modification. In SBR1, its relative gene frequency increased in SBR1 from 0.6 (day 349) to 4.4% (day 376). In SBR2, an increase from 0.7 to 5.7% (Fig. 4D) was observed although it occurred around 30 days after the beginning of Strategy III. The gene frequency of *Defluviicoccus* did not evolved in the same pattern and remained stable between 0.5 and 1.5% in SBR1, and between 0.5 and 2.5% in SBR2. An increase was nonetheless observed at the very end of the experiment in both reactors, but affected simultaneously and in similar proportion *Ca*. Competibacter.

Two common PAO in activated sludge, the beta-proteobacterium *Candidatus* Accumulibacter and the actinobacterial *Tetrasphaera*, evolved through a peculiar pattern. *Ca*. Accumulibacter was present in relatively high abundance at the beginning of the follow-up in both SBR while *Tetrasphaera* was barely detected (Fig. 4E and F). However, *Ca*. Accumulibacter seemed impacted by the cycle modification in SBR2 and its relative gene frequency dropped from 2.1% to 0.1% around 30 days after the start of Strategy III. Interestingly, it corresponded precisely to the increase of *Ca*. Competibacter. This seems in accordance with previous reports that identified an exclusion behavior between these bacteria due to a competition for the same substrate^23^. Additionally, the influent composition was relatively poor in TP and this may have participated to the out-competition of *Ca*. Accumulibacter by *Ca*. Competibacter^24^. However, this pattern was not observed in SBR1 and the initial population of *Ca*. Accumulibacter decreased from 1.4% (day 300) to 0.1% (day 313) despite a *status quo* in GAO abundance. It should be noted that the drop of *Ca*. Accumulibacter abundance was observed at the same time in both reactors (Fig. 5B). Therefore, a common cause might have trigged this phenomenon and it is reasonable to consider either the climatic conditions (mostly temperature) or wastewater composition. Two hypotheses can be put forward: a sudden increase of the COD:P ratio observed in the wastewater (Supplementary Figure S3) or a slow but constant drop of temperature with the coming of winter (this event occurred in late October). After the loss of *Ca*. Accumulibacter population, its initial abundance could not be restored until the end of the experiment. The absence of PAO lasted in both reactors until day 376 with the development of *Tetrasphaera* (Fig. 4E and F).

At the end of Strategy III, the filamentous bulking was solved in SBR1 and SBR2 and a significant population of *Ca*. Competibacter was established. The aggregates were dense and the SVI was good and stable. The main characteristics of the plant before and after the densification strategy revealed an interesting reduction of the specific energy demand for aeration from 1.06 to 0.74 kwh kg^−1^ COD_in bio_ while the volumetric load and the biological sludge production were kept unchanged (Table 2). The mean sludge retention time was higher after the densification strategy due to the development of denser aggregates and the biomass concentration in the reactors was more stable.

**Table 2:** Comparison of the main characteristics of the plant before (I) and after the densification strategy (III).

Parameter	Unit	Value	
		Conventional I	Densification III
Sludge retention time	d	24–37	29–54
Total suspended solids	kg m^−3^	2.7–3.6	3.2–3.8
Sludge loading total^a^	kg COD (kg TSS d)^−1^	0.150	0.102
Sludge loading biological^b^	kg COD (kg TSS d)^−1^	0.232	0.233
Biological sludge production	kg DW (kg COD_in WWTP_)^−1^	0.237	0.238
Volumetric load	m^3^ (m^3^_SBRs_ d)^−1^	0.295	0.248
Specific energy demand for aeration	kwh (kg COD_in bio_)^−1^	1.06	0.74

^a^Based on residual COD after the dissolved air flotation unit (kg day^−1^) over the total biomass present in both SBRs

^b^Based on the aeration time

In conclusion, this long-term full-scale study confirms the applicability of a periodic starvation to (1) solve recurring *Thiothrix* bulking in dairy WWTP, (2) convert loose aggregates into dense and compact granular-like structures and (3) considerably reduce energy demand for aeration.

## Materials and Methods

### Description of the plant

The investigated full-scale WWTP treated the process water of a dairy factory (Fig. 1). The range of manufactured products included milk, cream, butter, ice cream, cheese and yoghourt. The wastewater also comprised all liquid wastes coming from the cleaning. The preliminary treatment consisted of a fine screening to prevent damages and clogging of downstream equipment and piping. The influent was then collected in an equalization buffer tank (900 m^3^). During production, a second buffer tank (200 m^3^) received a continuous flow (~50 m^3^ h^−1^) from a DAF unit that removed about half of the COD content. The stream was then treated with two conventional SBRs operated in parallel (2 × 1,640 m^3^). The pre-treated wastewater characteristics are given in Table 3. A typical SBR cycle lasted eight hours and consisted of (1) simultaneous feed and mixed aeration, (2) aerobic reaction, (3) settling, (4) decanting and (5) sludge purge. The aerobic reaction step initially involved the addition of a metal salt for the precipitation of phosphorus. The DO concentration was maintained between 1.4 and 4.0 mg L^−1^. The designed sludge loading rate was 0.2 kg COD kg^−1^ TSS d^−1^. Before being discharged in the receiving watercourse, the biologically treated wastewater was post-treated with a drum filter to keep the TSS concentration below the required limits.

**Table 3:** Average value of the main parameters of the pretreated dairy wastewater (SBR influent).

Parameter	Mean (±SD)	Unit
Flow	870 (±255)	m^3^ day^−1^
Total COD	1358 (±485)	mg L^−1^
Soluble COD	868 (±447)	mg L^−1^
Total Nitrogen	55 (±5)	mg L^−1^
Total Phosphorus	11 (±5)	mg L^−1^
NH_4_^+^-N (dissolved)	9 (±13)	mg L^−1^
Temperature	24 (±3)	°C
pH	7.6 (±0.2)	—

### Long-term management of full-scale SBRs

In 2015, both SBRs frequently experienced losses of activated sludge due to filamentous bulking sludge phenomena. An experimental approach was set-up and divided into three mitigation strategies (Table 1). The first strategy was non-specific and consisted of occasional poly-aluminium chloride addition to mitigate the abundance of filamentous bacteria (Strategy I). From June 2015 (day 166), two specific control strategies were studied. One specific strategy aimed at reducing the volatile fatty acids (VFA) production in buffer tanks by increasing the oxidative redox potential (ORP) through the installation of aeration diffusers (Strategy II). Based on previous lab-scale experiments^12^, the other specific strategy (Strategy III) investigated the impact of the SBR feeding pattern by implementing either an aerated fill or an anaerobic fill. This approach explored the feasibility to select floc-forming microorganisms able to convert VFA into storage biopolymers, notably phosphate accumulating organisms (PAO) and glycogen accumulating organisms (GAO) by applying an extended anaerobic feeding mode, which enabled lysis and fermentation of particulate organic substrates into VFA.

Reactor performances were recorded on a daily (working day) basis by sampling the wastewater after the DAF treatment and after the drum filter polishing unit (Fig. 1). Phosphate, ammonium and nitrate were measured spectrophotometrically by the use of standard test kits (Hach-Lange). Total nitrogen, COD and suspended solids (total and volatile) were analyzed according to standard procedures. Sludge volume index (SVI_30_) were determined by reading the volume of the settled bed in a column after 30 min settling and calculated from the dry weight in mixed liquor suspended solids (MLSS). An additional dilution step of MLSS was performed prior to settling if necessary. Microscopic observations were performed with a Leica photonic microscope. The morphology of filaments and flocs was evaluated on a week-to-week basis.

### Quantitative PCR

Quantitative PCR (qPCR) were performed on sludge collected in SBR1 and SBR2 on a regular basis (see Fig. 5 in Results and Discussion section). Total genomic DNA was extracted with the PowerSoil^®^ DNA isolation kit (MoBio Laboratories) following manufacturer’s instructions. qPCR analysis were performed using primers targeting 16S rRNA gene from *Ca*. Accumulibacter (517 F and PAO846)^25, 26^ and *Thiothrix eikelboomi* (21Nf and 21Nr)^27^. For each analysis, a 7-point decimal calibration curve was established with solutions containing a known concentration of the amplicon generated by end-point PCR with the same primer pair. DNA concentration of the purified PCR product was measured with the QuantiFluor® dsDNA System (Promega) and was subsequently converted in quantity of fragments per unit of volume. Samples were measured in duplicates. qPCR was conducted on a CFX Connect Real-Time PCR Detection System (BioRad). Volumes of 20 μl of reaction mix were prepared with 10 μl GoTaq® qPCR Master Mix (Promega), 0.3 μM of both primers and 1 μl of DNA extract. The qPCR program was constructed with an activation step of 2 min at 95 °C, followed by 40 cycles of denaturation for 15 s at 95 °C and annealing/extension for 1 min at 60 °C. A melt curve was performed immediately after the last amplification cycle by increasing the temperature by 0.5 °C every 5 s up to 95 °C. Data processing was performed on CFX Manager 3.0 software (BioRad). Target-specific 16S rRNA gene quantification was expressed as number of copies of 16S rRNA operon per mg TSS.

### 16S amplicon sequencing

The bacterial diversity was assessed in sludge samples collected in SBR1 and SBR2 by sequencing the V1 and V2 hypervariable regions of the 16S rRNA gene on Illumina MiSeq. The sequencing and pipeline processing was performed at Research and Testing Laboratory (Lubbock, USA). The number of reads per sample ranged from 16 570 to 61 540. The forward primer was constructed with the Illumina i5 adapter, an 8–10 bp barcode and primer 28 F (5′-AGTTTGATCNTGGCTCAG-3′). The reverse primer was constructed with the Illumina i7 adapter, an 8–10 bp barcode and primer 388 R (5′-TGCTGCCTCCCGTAGGAGT-3′). Amplifications were performed with Qiagen HotStar Taq master mix and 0.25 μM of each primer. Reactions were performed on ABI Veriti thermocyclers (Applied Biosytems) under the following thermal profile: 95 °C for 5 min, then 35 cycles of 94 °C for 30 sec, 54 °C for 40 sec, 72 °C for 1 min, followed by one cycle of 72 °C for 10 min and 4 °C hold. Amplification products were pooled equimolar. Each pool was size selected using Agencourt AMPure XP (BeckmanCoulter) in a 0.7 ratio for both rounds. Size selected pools were quantified using the Qubit 2.0 Fluorometer (Life Technologies) and loaded on an Illumina MiSeq 2 × 250 flow cell at 10 pM.

Forward and reverse reads were merged together using the PEAR Illumina paired-end read merger^28^. Reads were trimmed and sorted from longest to shortest. Then, a prefix de-replication was performed using the USEARCH algorithm^29^. A clustering at a 4% divergence was performed using USEARCH clustering algorithm. Singleton clusters were removed at this stage. Operational Taxonomic Unit (OTU) selection was performed with the UPARSE OTU selection algorithm^30^ to classify clusters into OTUs. Chimera checking was performed using the UCHIME chimera detection software^31^ executed in *de novo* mode. Each centroid was mapped to its corresponding OTU and tagged as chimeric or non-chimeric. USEARCH global alignment algorithm^29^ was then used to map each read to its corresponding non-chimeric cluster. Finally, a correction step was performed between each sequence of a cluster and the consensus sequence of that cluster. Sequences were then matched against a database of high quality sequences derived from NCBI. The sequence data have been submitted to the NCBI Sequence Read Archive (SRA) (http://www.ncbi.nlm.nih.gov/sra) under the accession number SRP095156. The abundance of taxa was expressed as “relative gene frequency”. This parameter corresponded to the number of reads of a given taxon in a sludge sample divided by the total number of reads in the same sludge sample (expressed in percent).

## Electronic supplementary material

Supplementary material
