# Supplementary material for: Filamentous bulking caused by Thiothrix species is efficiently controlled in full-scale wastewater treatment plants by implementing a sludge densification strategy
Source: Sci Rep. 2017 May 3;7:1430. doi: 10.1038/s41598-017-01481-1 (PMC5431194; doi:10.1038/s41598-017-01481-1)
Supplement: Supplementary file 1 — Supplementary material [file 41598_2017_1481_MOESM1_ESM.pdf]

Supplementary material for

Filamentous bulking caused by *Thiothrix* species is efficiently controlled in full-scale wastewater treatment plants by implementing a sludge densification strategy

Olivier Henriet[1°], Christophe Meunier[2°*], Paul Henry[2] and Jacques Mahillon[1]

°These authors contributed equally to this work

[1]Laboratory of Food and Environmental Microbiology, Université catholique de Louvain, Louvain-la-Neuve, Belgium

[2]CEBEDEAU, Research and expertise center for water, Allée de la découverte, 11 (B53), Quartier Polytech 1, B-4000 Liège, Belgium

*Corresponding author:

Christophe Meunier

CEBEDEAU, Research and expertise center for water

Allée de la découverte 11 (B53), Quartier Polytech 1

B-4000 Liège

Email: cmeunier@cebedeau.be

Phone: +32 (0)4 252 12 33

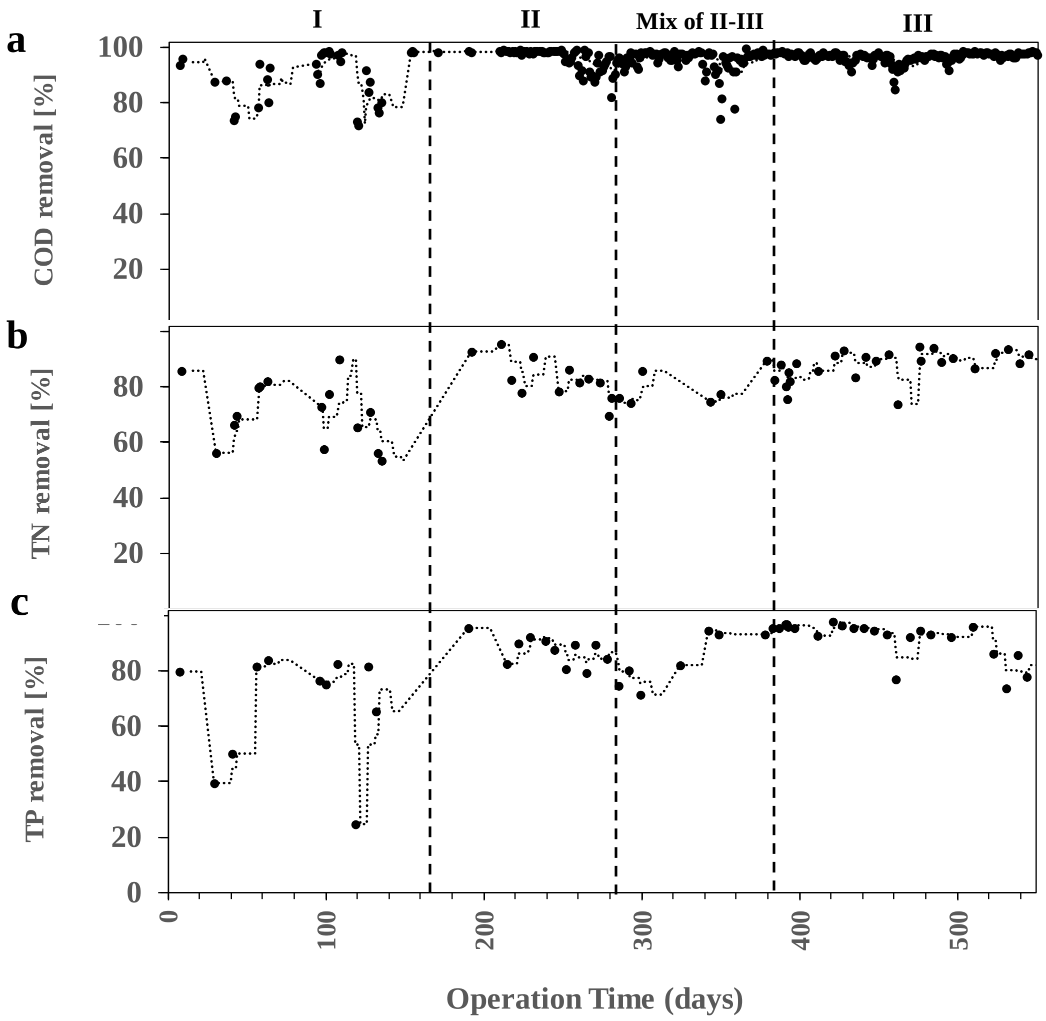

Supplementary Figure S1. Overall performances of the SBRs coupled with a drum-filtration unit. (a) COD removal efficiency, (b) total nitrogen (TN) removal efficiency and (c) total phosphorus (TP) removal efficiency under three different strategies: addition of polyaluminium chloride (I), inhibition of VFA production (II) and densification by periodic starvation (III). The transition period from day 282 to day 358 (II-III) corresponds to a combination of the inhibition of VFA production in the buffer tank with or without a modification of the feeding pattern of the SBRs. The post-treatment filtration was deactivated from day 418 to day 461.

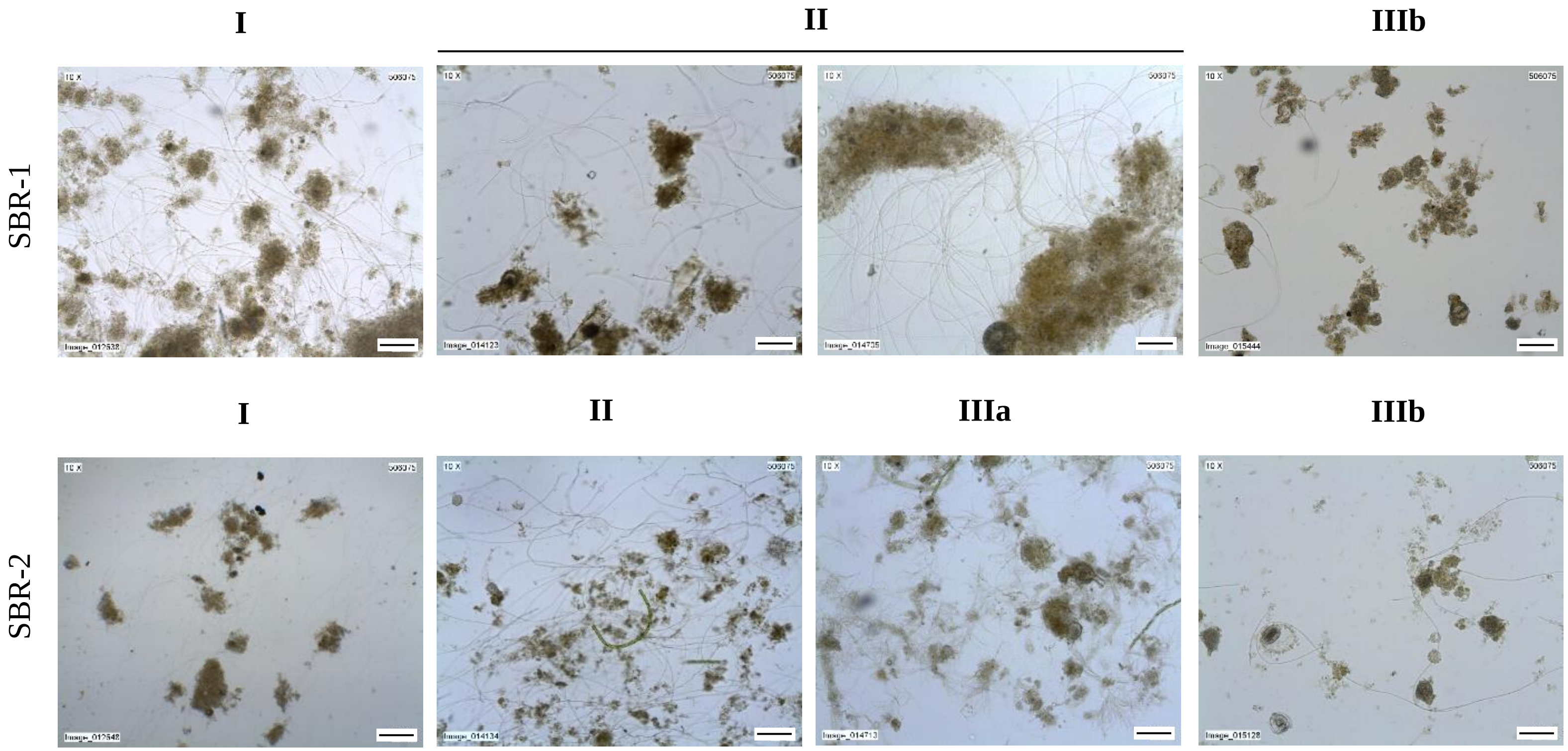

Supplementary Figure S2. Morphological evolution of the sludge. Optical micrographs of biomass in SBR1 and SBR2 under three mitigation strategies consisting in the addition of polyaluminium chloride (I), inhibition of VFA production (II) and modification of the feeding pattern with (IIIa) or without (IIIb) aeration in the buffer tanks. The pictures were obtained during the last month of each operation period (scale bar corresponds to 100 microns).

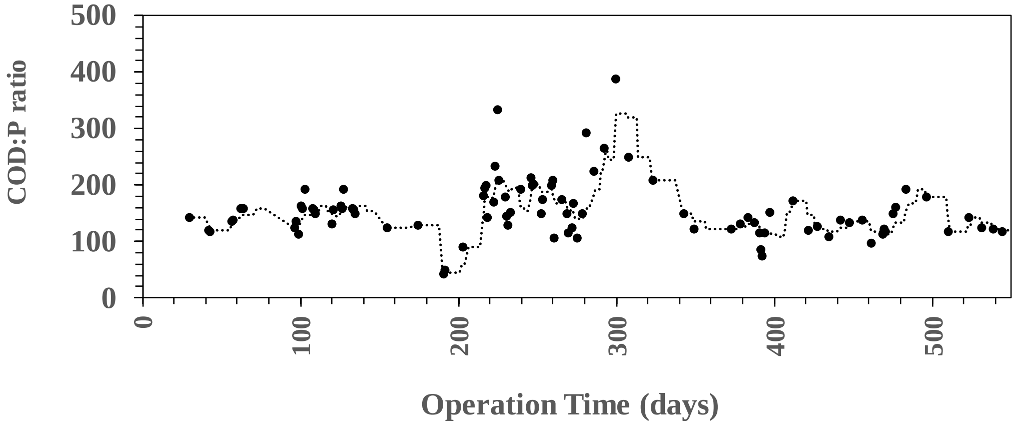

Supplementary Figure S3. COD:P ratio of the pretreated dairy wastewater (SBR influent) over the experimental period.
